# Variation in the hatching rate of larvae of four tick species from laboratory colonies

**DOI:** 10.1590/S1984-29612025055

**Published:** 2025-10-13

**Authors:** Ygor Henrique da Silva, Marisa Beatriz da Silva Rocha, Ester Oliozi Marré, Manuela Pimentel da Motta, Brena Gava Guimarães, Thais Ribeiro Correia Azevedo, Guilherme Marcondes Klafke, Barbara Rauta de Avelar, Diefrey Ribeiro Campos, Fabio Barbour Scott

**Affiliations:** 1 Universidade Federal Rural do Rio de Janeiro – UFRRJ, Instituto de Veterinária – IV, Departamento de Parasitologia – DPA, Seropédica, RJ, Brasil; 2 Instituto de Pesquisas Veterinárias Desidério Finamor – IPVDF, Eldorado do Sul, RS, Brasil

**Keywords:** Colony, ixodids, egg viability, Colônia, ixodideos, viabilidade de ovos

## Abstract

The objective of this study was to evaluate the hatching percentage of tick larvae of *Amblyomma sculptum*, *Dermacentor nitens*, *Rhipicephalus linnaei*, and three strains of *Rhipicephalus microplus*. The egg masses laid by females of each tick species were weighed in different amounts (125, 250 and 500mg), placed in adapted 3 and 5 mL syringes, and incubated at 27°C and 80% relative humidity for 25 days for *A. sculptum* and 21 days for the remaining species. After this period, larval hatching rate was evaluated and data were analyzed through correlation and comparison between groups. The results showed that the average hatching rates varied from 27.9% to 42.2% for *A. sculptum*, 86.6% to 87.7% for *D. nitens*, 74.4% to 80.0% for *R. linnaei*, and *R. microplus*: 75.3% to 82.6% (UFRRJ strain), 64.6% to 72.2% (Mozo strain), and 71.8% to 75.2% (Porto Alegre strain). Although statistically significant differences were observed in the mean percentages between groups, the correlation was weak. We concluded that the weight of the egg masses and the volume of the containers did not significantly affect larval hatching.

## Resumo

O objetivo do estudo foi avaliar o percentual de eclosão de larvas de carrapatos das espécies de *Amblyomma sculptum*, *Dermacentor nitens*, *Rhipicephalus linnaei* e três cepas de *Rhipicephalus microplus*. A oviposição das fêmeas de cada espécie foi pesada em diferentes medidas (125, 250 e 500 mg), acondicionada em seringas adaptadas de 3 e 5 mL e as seringas foram incubadas a 27°C e 80% de umidade relativa por 25 dias para *A. sculptum* e 21 dias para as demais espécies. Após esse período foi avaliado o percentual de eclosão de larvas, com os dados analizados por correlação e por comparação entre os grupos. Como resultados, foi observado que os percentuais médios de eclosão entre os grupos variaram de 27,9 a 42,2% para *A. sculptum,* de 86,6 a 87,7% para *D. nitens*, de 74,4 a 80,0% para *R. linnaei* e para *R. microplus* de 75,3 a 82,6% (cepa UFRRJ), 64,6 a 72,2% (cepa Mozo) e 71,8 a 75,2% (cepa Porto Alegre). Apesar das diferenças estatísticas dos percentuais médios entre os grupos, estes tiveram uma fraca correlação. Conclui-se que o peso das massas de ovos e o volume dos recipientes não interferiram significativamente na eclosão das larvas.

## Introduction

Ticks of the family Ixodidae (Acari: Parasitiformes) are hematophagous ectoparasites that affect a wide range of hosts, including humans. Among the species of the Ixodidae family, *Amblyomma sculptum*, *Dermacentor nitens*, *Rhipicephalus microplus* and *Rhipicephalus linnaei* are the main ticks reported parasitizing pets and humans (Otranto et al., 2021; Nogueira et al., 2022). The importance of these ectoparasites lies in the skin lesions and hypersensitivity reactions caused by salivary secretions during blood feeding (Barros-Battesti et al., 2006; Rodrigues et al., 2010; Barbosa et al., 2023) and also because they act as vectors of pathogens relevant to One Health, such as *Rikettsia rickettsii*, the agent of Brazilian Spotted Fever, a disease with high human lethality, transmitted by *A. sculptum* and, in some regions, by *R. linnaei* (Almeida et al., 2013; Dantas-Torres et al., 2024). *Ehrlichia canis* and *Babesia vogeli*, which cause canine ehrlichiosis and babesiosis, respectively, transmitted by *R. linnaei*. Furthermore, this tick is common in peri-urban environments, increasing the risk of human infestation (Nogueira et al., 2022; Dantas-Torres et al., 2024). *Rhipicephalus microplus*, a vector of *Babesia bovis*, *Babesia bigemina*, *Anaplasma marginale* and *Theileria equi*, and *D. nitens*, is associated with the transmission of *Babesia caballi*, negatively interfering with the productivity of cattle and horses. Annual economic losses related to the treatment and control of these tick infestations are estimated at R$ 17.6 billion (Díaz-Sánchez et al., 2020; Barros et al., 2024).

Given their significance, ticks have been the focus of research aimed at better understanding their biological characteristics and vector capacity and developing new control methods. The use of synthetic and natural chemicals with acaricidal activity remains the primary strategy employed to manage infestations (Otranto et al., 2021; Obaid et al., 2022). However, the development and approval of these compounds require rigorous testing conducted both *in vitro* and clinical efficacy studies using animal models (Otranto et al., 2021). Therefore, establishing and maintaining tick colonies is essential not only for conducting these tests but also for advancing studies related to physiology, biochemistry, and other aspects of the biology of these arthropods (Borges et al., 1999; Calligaris et al., 2013; Labruna & Faccini, 2020).

Studies on tick biology have reported the influence of various biotic (such as host species and predators, such as fungi, bacteria, and nematodes) (Daemon et al., 1998; Cafarchia et al., 2015; Ma et al., 2016) and abiotic factors (including temperature, humidity, photoperiod, and gas concentration) (Despins, 1992; Bellato & Daemon, 1997; Labruna, 2018; Labruna & Faccini, 2020; Sales et al., 2024) that affect their development. However, even under controlled biotic and abiotic conditions, variable results are sometimes observed for biological parameters compared to those reported in the literature. These include differences in the molting rate from immature stages to adulthood, female weight, weight and shape of egg masses, and larval hatching rate (Dipeolu, 1991; Despins, 1992; Serra-Freire & Furlong, 1993; Ma et al., 2016; Labruna & Faccini, 2020).

The hatching rate of tick larvae is a crucial parameter in the evaluation of biological and control studies, particularly for *in vitro* and *in vivo* testing of ectoparasiticides (Drummond et al., 1969; Figueiredo et al., 2018). Although some publications report varying hatching rates for larvae of different tick species (Szabó et al., 2009; Ma et al., 2016; Labruna, 2018; Labruna & Faccini, 2020; Sales et al., 2024), no one has thoroughly assessed a colony established over an extended period. In this context, it is essential to investigate colonies maintained long-term to better understand variations in hatching rates and to enhance control strategies for these ectoparasites. Therefore, this study aimed to evaluate variation in larval hatching rates of the tick species *A. sculptum*, *D. nitens*, *R. microplus* (UFRRJ, Mozo, and Porto Alegre strains), and *R. linnaei* maintained in laboratory colonies.

## Materials and Methods

### Study location

This study was conducted at the Laboratory of Experimental Chemotherapy in Veterinary Parasitology (LQEPV), part of the Department of Animal Parasitology at the Veterinary Institute of the Federal Rural University of Rio de Janeiro (UFRRJ), located on the Seropédica Campus, Rio de Janeiro, Brazil.

### Origin of the ticks

The ticks used in this study originated from laboratory colonies of *A. sculptum*, *D. nitens*, *R. microplus* and *R. linnaei*. Ticks were collected following the protocols used to maintain the laboratory colony. For the heteroxenic species *A. sculptum* and *R. linnaei*, naive rabbits were artificially infested after fixing fabric hoods with Una glue (Neitz et al., 1971) to the back and ears, respectively. The animals used to maintain the *A. sculptum* colony were infested with 100 unfed couples aged approximately 35 days; the engorged females were recovered on the 10th day after natural detachment. While those used to maintain the *R. linnaei* colony were infested with 200 couples aged approximately 21 days; the engorged females were recovered on the seventh day after natural detachment.

Regarding monoxenic species, the animals were kept individually stabled in pens lined with wooden platforms for daily recovery of engorged females that were naturally released. To maintain the *R. microplus* colony of the UFRRJ strain, crossbred cattle with natural infestations were stabled for 21 days for recovery of engorged females, after which they were replaced by other animals also with natural infestations. While the colonies of *R. microplus* strains Porto Alegre and Mozo, crossbred cattle weighing up to 200 kg, sensitive to tick infestation, were artificially infested up to three times a week on alternate days with 10,000 to 40,000 unfed larvae at approximately 14 to 21 days of age. Regarding the *D. nitens* colony, ponies were artificially infested twice a week with 1800 to 5400 larvae aged between 14 and 30 days. The animals used for artificial infestations were kept in stables for a period of no more than three months and the recovery of engorged females was performed daily during this period from the 21st day after the first infestation.

The sources and protocol numbers approved by the Animal Ethics Committee of the Universidade Federal Rural do Rio de Janeiro are detailed in Table 1.

**Table 1:** Origin data of the colonies of *Amblyomma sculptum*, *Dermacentor nitens*, *Rhipicephalus microplus*, and *Rhipicephalus linnaei*, along with their respective approval numbers from the Animal Ethics Committee.

**Tick Species**	**Collection host**	**Locality**	**Date of Colony Establishment**	**Maintenance host**			**CEUA Protocol**
				**Species**	**Breed**		
*Amblyomma sculptum*	Horses	Seropédica, RJ, Brazil	February, 2015	Rabbits (*Oryctolagus cunniculus*)		New Zealand	1268101223
*Dermacentor nitens*	Horses	Seropédica, RJ, Brazil	March, 2015	Horses (*Equus caballus*)		Brazilian Pony	4653140723
*R. microplus* UFRRJ strain	Cattle	Seropédica, RJ, Brazil	- - -	Cattle *(Bos taurus x Bos indicus)*		*Crossbreed*	2787140723
*R. microplus* Mozo strain	Cattle	Uruguay	April, 2014				
*R. microplus* Porto Alegre strain	Cattle	Porto Alegre, Brazil	September, 2018				
*Rhipicephalus linnaei*	Dogs	Seropédica, RJ, Brazil	January 1999	Rabbits (*Oryctolagus cunniculus*)		New Zealand	9812271021

CEUA = Ethics Committee on Animal Use - UFRRJ; RJ = Rio de Janeiro.

### Incubation of ticks

From March 2023 to March 2024, females of the four tick species and strains maintained in laboratory colonies recovered after feeding and spontaneously detaching from their maintenance hosts (Table 1). The ticks were then washed in running water, dried with absorbent paper and placed in Petri dishes (90 mm × 15 mm) in groups of up to 20 females with a weight range of 500 to 900 mg for the species *A. sculptum*, 50 females with 260 to 440 mg for *D. nitens* and 60 females with 160 to 360 mg and 120 to 250 mg for *R. microplus* (all strains) and *R. linnaei*, respectively, per plate. They were stored in climate-controlled chambers at 27 ± 1°C and 80 ± 10% relative humidity for 25 days for *A. sculptum* (as described by Travassos & Vallejo-Freire, 1944, and Serra-Freire & Furlong, 1993) and for 21 days for the other species (as described by Drummond et al., 1969 and Koch, 1982).

After this period, the egg masses laid by these females were pooled and homogenized in a single dish according to species/strain and incubation date and then separated into aliquots of 125, 250, and 500 mg. The portions were placed in 3 mL or 5 mL plastic syringes, and the tips were removed and sealed with cotton. The syringes were then incubated in a climate-controlled chamber under the same conditions and periods described above. Each group had five replicates per combination of egg mass and syringe volume, totaling 300 syringes for each group, species, and strain.

### Hatching analysis

For larval hatching analysis, the method proposed by Figueiredo et al. (2018) was used. After the incubation period, the syringes and their contents (infertile eggs, eggshells, and hatched larvae) were kept at -20°C for 24 hours for inactivation. The contents of each syringe were then homogenized using an entomological stylus, and a 50 mg aliquot was placed onto Petri dishes (60 mm × 15 mm) filled with 70% ethanol. Using a stereomicroscope and a cell counter (Kacil® model CCS-01), 100 individuals (eggs or larvae) were counted. Hatching rate was calculated using the following equation: number of larvae/total number of individuals × 100.

### Data analyses

The descriptive analysis of larval hatching data for each tick species/strain was performed using Microsoft Excel®, calculating the observed mean and standard deviation. Before statistical analysis, both for assessing correlation and for comparison between groups, data distribution was assessed using the D'Agostino-Pearson test. Spearman’s correlation coefficient test was used to determine the correlation between larval hatching percentage, syringe volume, and incubated egg aliquot weight for each species/strain. Differences in hatching percentages among the egg mass/syringe volume groups were evaluated using ANOVA, preceded by t-tests for data with parametric distribution, and the Kruskal-Wallis test for data with non-parametric distribution. Analyses were conducted using BioEstat 5.3, assuming a 95% confidence level (p≤0.05).

## Results

**Figure 1:**
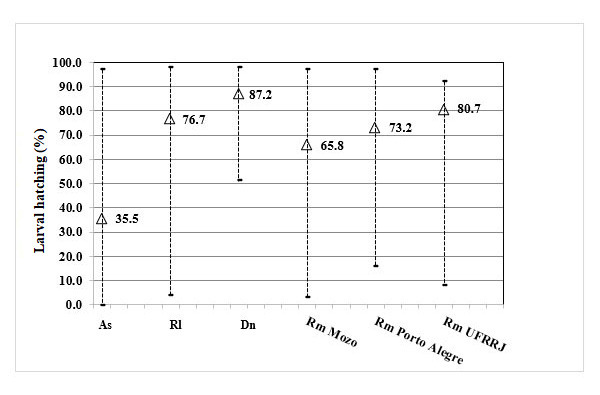
Larval hatching percentage of different tick species/strains maintained in a laboratory colony over one year. As = *Amblyomma sculptum*; Dn = *Dermacentor nitens*; Rl = *Rhipicephalus linnaei*; Rm = *Rhipicephalus microplus*.

Over the course of one year, more than 300 evaluations were conducted for each tick species to determine larval hatching rates. The mean larval hatching rates, followed by their respective standard deviations, were 87.2% (±6.3) for *D. nitens*, 80.7% (±14.3) for *R. microplus* UFRRJ strain, 76.7% (±11.9) for *R. linnaei*, 73.2% (±16.6) for *R. microplus* Porto Alegre strain, 65.8% (±15.2) for *R. microplus* Mozo strain, and 35.5% (±23.1) for *A. sculptum* (Figure 1).

Larval hatching percentages in relation to syringe size and egg mass weight are presented in Table 2.

**Table 2:** Verification of the influence of different egg mass weights and syringe volumes on the hatching percentage of *Amblyomma sculptum*, *Rhipicephalus linnaei*, *Dermacentor nitens* and *Rhipicephalus microplus* larvae (Mozo, Porto Alegre and UFRRJ strains) after incubation at 27 ± 1°C and 80 ± 10%.

**Tick species / Strain**			**Syringe Volumes / Egg Mass Weights**					
			**3 mL**			**5 mL**		
			**125 mg**	**250 mg**	**500 mg**	**125 mg**	**250 mg**	**500 mg**
*As*	**Larval hatching (%)**	Mean (SD)	33.3^aA^ (24.7)	41.6^bB^ (21.6)	42.6^bB^ (20.7)	27.9^aA^ (23.8)	33.8^aA^ (25.5)	34.2^aA^ (18.9)
		Min - Max	0 – 91	1 - 81	0 – 76	1 – 79	0 – 97	2 – 73
*Rl*		Mean (SD)	77.8^aA^ (13.7)	76.7^aA^ (15.4)	80.0^aA^ (9.7)	74.4^aB^ (11.5)	74.4^aB^ (9.4)	76.8^aB^ (10.3)
		Min - Max	42 – 98	4 – 95	60 – 97	47 –95	49 – 89	49 –97
*Dn*		Mean (SD)	87.1^aA^ (5.8)	87.6^aA^ (5.2)	87.7^aA^ (7.8)	86.6^aA^ (6.8)	87.1^aA^ (5.8)	86.8^aA^ (6.1)
		Min - Max	72 – 96	76 – 98	51 – 97	70 – 97	70 – 98	69 – 95
*Rm* Mozo		Mean (SD)	72.2^aA^ (18.1)	68.1^aA^ (15.1)	71.6^aA^ (12.4)	64.6^bB^ (16.3)	71.4^aA^ (14.7)	70.9^aA^ (12.4)
		Min - Max	9 – 97	28 – 95	44 – 92	3 – 90	22 – 92	22 –91
*Rm* Porto Alegre		Mean (SD)	75.2^aA^ (17.2)	71.9^aA^ (16.8)	71.8^aA^ (16.5)	73.7^aA^ (18.6)	74.1^aA^ (15.7)	73.0^aA^ (15.0)
		Min - Max	32 – 97	32 – 95	16 – 97	23 – 97	23 – 93	42 –96
*Rm* UFRRJ		Mean (SD)	82.5^aA^ (14.3)	82.5^aA^ (12.)2	82.6^aA^ (13.4)	79.1^aB^ (14.5)	75.3^aB^ (16.6)	81.9^aB^ (13.4)
		Min - Max	47 – 98	43 – 99	48 – 99	41 – 98	8 –96	47 – 96

As = *Amblyomma sculptum*; Dn = *Dermacentor nitens*; Rs = *Rhipicephalus linnaei*; Rm = *Rhipicephalus microplus*; SD = standart derivation; Min = minimum; Max = maximum. Lowercase letters indicate statistically significant differences at a 95% confidence level (*p*≤0.05) between weights of egg clutches within the same syringe volume. Uppercase letters indicate statistically significant differences at a 95% confidence level (*p*≤0.05) for the same egg clutch weight across different syringe volumes. (Analysis performed using ANOVA and Kruskal-Wallis tests.)

The mean hatching percentages among the evaluated groups for each tick species and strain were generally similar. However, statistical analysis revealed significant differences in syringe volume and egg mass weight between *A. sculptum* (p < 0.03), *R. linnaei* (p < 0.03), *R. microplus* UFRRJ strain (p < 0.02), and *R. microplus* Mozo strain (*p* < 0.04) (Table 2).

## Discussion

To our knowledge, this is the first study to evaluate larval hatching rates of different tick species maintained in laboratory colonies over a one-year period. Based on the results presented, the highest hatching rates were observed in one-host ticks such as *D. nitens*, with average rates exceeding 85% in all assessments performed in this study, consistent with those reported by Labruna & Faccini (2020) and Rodrigues et al. (2017), who also observed high average larval hatching rates, ranging from 87.9% to 95.5% under controlled temperature and humidity conditions.

Variations in the average larval hatching rate of *R. microplus* were observed among the strains analyzed. This difference may be related to the length of time during which the colonies were established. As discussed by de Oliveira & Alencar (1987), repeated infestations of successive generations in the same host can elicit different immunological responses, including reduced egg viability. This may explain the lower hatching rates observed in the Mozo and Porto Alegre strains than in the UFRRJ strain. The former two were maintained in animals subjected to artificial infestations over a period of approximately six months, whereas the UFRRJ strain consisted of first-generation ticks obtained from naturally infested, stable animals. When comparing the hatching percentages of the UFRRJ strain with those reported in the literature, the results were consistent, with average larval hatching rates ranging from 80.2 to 94.1% (Sales et al., 2024).

The lowest hatching rate observed in this study was recorded for *A. sculptum*, diverging from the findings of previous studies such as Chacón et al. (2003) and Sanavria & Prata (1996) which, under similar conditions to the current study, found larval hatching rates above 54%. However, compared to more recent results, the results of the present study could indicate species-specific biological characteristics that have changed over time. These results are consistent with those reported by Labruna (2018), who described the mean larval hatching rate in control groups of *A. sculptum* as an average of 29.3%, ranging from 5% to 95%. This low hatching rate has also been reported for other species of the genus *Amblyomma*. For example, Labruna (2018) reported a mean larval hatching rate of 23.8% (ranging from 0 to 70%) in *Amblyomma cajennense*. Similarly, Szabó et al. (2009) observed a hatching rate of only 1.2% for *Amblyomma incisum* under controlled laboratory conditions. However, when eggs were incubated in forest fragments, the hatching rate increased significantly to 72.2%, suggesting that natural environmental conditions may play a critical role in the hatching success of *A. incisum*.

For *R. linnaei*, larval hatching rates reported in the literature range from 53.9% to 97% for ticks whose engorged females were recovered from dogs (Dipeolu,1991; Bechara et al., 1995; Hadi & Adventini, 2015) and the mean observed in the present study, 76.7%, was within this range. In the case of *A. sculptum* and *R. linnaei*, it is important to note that the lower hatching rates may be associated with the use of alternative hosts for maintaining colonies in the laboratory, given that these are not considered primary hosts for these species. Although there are many studies demonstrating satisfactory results of ecdysis, engorgement and reproduction in colonies of ixodid ticks (Sanavria & Prata 1996; Bellato & Daemon, 1997), there are also others that report changes in the size and number of engorged stages recovered, as well as changes in the shape of eggs from engorged females (Dipeolu, 1991; Ma et al., 2016; Rodrigues et al., 2017). However, for the maintenance of tick colonies with three hosts, the use of rabbits as hosts is a more practical alternative, mainly due to the different detachment times required for ecdysis or oviposition in the environment (Paz et al., 2008).

Although it was initially hypothesized that egg mass and/or available space within the syringes could influence larval hatching, the results showed only weak correlations. This suggests that the observed variation in hatching rates is likely part of the natural biological variability of these tick species rather than a direct effect of this variable. Similar variations have been reported in other studies that used different incubation containers. For instance, Serra-Freire & Ahid (1993) used nylon fabric envelopes to incubate *D. nitens* eggs and observed hatchability ranging from 60% to 100%. Labruna & Faccini (2020), studying both field and laboratory populations of *D. nitens* in glass tubes, reported hatching rates from 22% to 93% and from 87% to 99%, respectively. Ma et al. (2016) observed different hatching averages for *R. microplus* depending on the host: 83.0% when fed on rabbits, 96.0% on cattle, and only 19.2% on sheep. These findings indicate that larval hatchability may be strongly influenced by biotic factors, including insufficient female feeding (Travassos & Vallejo-Freire, 1944) and the host species used for tick development (Ma et al., 2016). These factors can affect egg viability, leading to infertility or desiccation of eggs, which was also observed during the study’s hatchability assessments (Oliveira & Alencar, 1987). Additionally, abiotic factors, including temperature, humidity, and gas saturation in the environment, can also play a crucial role in embryonic development (Despins, 1992; Szabó et al., 2009; Labruna & Faccini, 2020).

Another variable that may influence hatchability is the evaluation method. The method of counting eggs, the criteria used to classify viable eggs, and the selection of data analyzed can all affect the results (Serra-Freire & Ahid, 1993; Figueiredo et al., 2018; Labruna & Faccini, 2020). Some studies report only data from samples considered “normal,” excluding atypical outcomes which may obscure the true biological variability of the species and lead to misinterpretation of the dynamics of these parasites.

Based on the findings presented here, it is evident that larval hatching rates in tick populations can vary, even under controlled conditions (Hadi & Adventini, 2015; Rodrigues et al., 2017; Labruna, 2018). This variation appears to be primarily linked to the biological and adaptive characteristics inherent to each species, as well as to factors that are difficult to control, including the host immune response, infections by entomopathogenic organisms, and incomplete feeding by engorged females (Travassos & Vallejo-Freire, 1944).

Although the establishment and maintenance of a colony are crucial to control variables and maintain the sensitivity of the organism, the length of time the colony exists can pose challenges. Over time, there may be a gradual loss of genetic variability and population vigor, directly impacting its evolutionary and reproductive functions. For this reason, and as recommended by the World Association for the Advancement of Veterinary Parasitology (WAAVP), the existence of very old colonies is not recommended (Holdsworth et al., 2022). Therefore, the responsible and controlled insertion of new specimens becomes essential to stimulate genetic prediction and population health.

Despite these influences, the data suggest that, even under optimal laboratory conditions, hatching rates below 90% are common and do not compromise parasite survival or the maintenance of laboratory colonies. However, for *in vitro* studies, a larval hatching rate above 80% is generally preferable. In addition, no consistent influence of incubation volume and egg mass weight on hatching rates was observed. Therefore, these parameters can be adjusted according to the routine and operational needs of each laboratory without negatively affecting outcomes.

## Conclusion

Based on the results obtained in this study, it can be concluded that under laboratory conditions, the average hatching rate was approximately 35% for *A. sculptum* and ranged from 65% to 90% for *D. nitens*, *R. microplus*, and *R. linnaei*. Additionally, neither egg mass nor storage volume significantly affected larval hatching rates.

## Data Availability

Data will be made available on request.
